# Tunable short-wavelength spin wave excitation from pinned magnetic domain walls

**DOI:** 10.1038/srep21330

**Published:** 2016-02-17

**Authors:** Ben Van de Wiele, Sampo J. Hämäläinen, Pavel Baláž, Federico Montoncello, Sebastiaan van Dijken

**Affiliations:** 1 Department of Electrical Energy, Systems and Automation, Ghent University, Technologiepark 913, B-9052 Ghent, Belgium; 2 NanoSpin, Department of Applied Physics, Aalto University School of Science, P.O. Box 15100, FI-00076 Aalto, Finland; 3 Department of Condensed Matter Physics, Charles University, Ke Karlovu 5, 121 16 Prague, Czech Republic; 4 Dipartimento di Fisica e Scienze della Terra, Università degli Studi di Ferrara, Via Saragat 1, 44122 Ferrara, Italy

## Abstract

Miniaturization of magnonic devices for wave-like computing requires emission of short-wavelength spin waves, a key feature that cannot be achieved with microwave antennas. In this paper, we propose a tunable source of short-wavelength spin waves based on highly localized and strongly pinned magnetic domain walls in ferroelectric-ferromagnetic bilayers. When driven into oscillation by a microwave spin-polarized current, the magnetic domain walls emit spin waves with the same frequency as the excitation current. The amplitude of the emitted spin waves and the range of attainable excitation frequencies depend on the availability of domain wall resonance modes. In this respect, pinned domain walls in magnetic nanowires are particularly attractive. In this geometry, spin wave confinement perpendicular to the nanowire axis produces a multitude of domain wall resonances enabling efficient spin wave emission at frequencies up to 100 GHz and wavelengths down to 20 nm. At high frequency, the emission of spin waves in magnetic nanowires becomes monochromatic. Moreover, pinning of magnetic domain wall oscillators onto the same ferroelectric domain boundary in parallel nanowires guarantees good coherency between spin wave sources, which opens perspectives towards the realization of Mach-Zehnder type logic devices and sensors.

Collective spin wave excitations in ferromagnetic materials provide a promising platform for low power wave-like computing in nanoscale devices up to THz frequencies^1^,^2^,^3^. Several concepts and building blocks for spin wave technologies have been developed in recent years, including magnonic crystals^4^,^5^, logic gates^6^,^7^,^8^, transistors^9^, and multiplexers^10^. Spin wave emission is an essential part of all these structures. Electrical excitation of spin waves in ferromagnetic materials is conventionally realized by the use of a microwave antenna. One of the drawbacks of this approach is the impossibility to efficiently excite spin waves with wavelengths that are shorter than the antenna width^11^, limiting the downscaling of devices. Motivated by this shortcoming, alternative approaches for the emission of short-wavelength spin waves have been explored recently. Examples include the design of wavelength conversions in the vicinity of microwave antennas using tapered waveguides^12^ or magnetic grating couplers^13^, the use of resonantly driven microwave-to-spin-wave transducers^14^, and the injection of spin waves from spin-torque nano-oscillators^15^,^16^,^17^,^18^. Topological defects in ferromagnetic films such as vortices and domain walls provide another attractive route towards the realization of localized spin-wave sources^19^. When brought into oscillation, the non-collinear spin structure of a defect induces regular magnetic perturbations that propagate through the ferromagnetic layer as spin waves. If the lateral size of the topological defect is small and its oscillation amplitude is restricted by a strong pinning potential, the wavelength of the emitted spin waves can be extremely short.

In this work, we propose a highly tunable source of short-wavelength spin waves based on strongly pinned oscillating domain walls in a ferromagnetic-ferroelectric bilayer. In particular, we consider ferromagnetic films that are strain-coupled to a ferroelectric BaTiO_3_ layer, but the concept can be extended to other ferroelectric materials and magnetoelectric coupling mechanisms. Strain-coupling between the ferroelectric and ferromagnetic layer originates in the transfer of in-plane lattice tetragonality from the ferroelectric domains to the ferromagnetic material, leading to modulations of magnetic anisotropy via inverse magnetostriction. While other domain patterns are also suitable, the ferroelectric layer considered in this paper consist of a regular repetition of *a*_1_- and *a*_2_ stripe domains. This domain pattern is characterized by abrupt 90 degree rotations of the ferroelectric polarization and lattice tetragonality in the film plane. Because of strain coupling, uniaxial magnetic anisotropy is induced in the ferromagnetic film that covers the alternating *a*_1_- and *a*_2_ stripe domains. The orientation of the uniaxial magnetic anisotropy follows the lattice tetragonality in the ferroelectric layer, i.e. it rotates by 90 degrees at domain boundaries. The regular modulation of magnetic anisotropy in the ferromagnetic film leads to full domain pattern transfer from the ferroelectric layer, an effect that has been experimentally demonstrated for various magnetic materials on BaTiO_3_^20^,^21^,^22^,^23^. A schematic illustration of the ferroelectric-ferromagnetic bilayer in zero magnetic field is shown in Fig. 1. Besides the formation of regular magnetic stripe domains, strong magnetic domain wall pinning on top of perfectly straight ferroelectric domain boundaries is another key attribute of the hybrid material system. Since the ferroelectric boundaries are only a few nanometers wide^24^,^25^, the strain-induced 90 degree rotations of uniaxial magnetic anisotropy occur nearly instantly. This effect leads to strong and highly localized pinning of straight magnetic domain walls and enables the emission of short-wavelength spin waves.

To analyze spin wave excitation from a pinned magnetic domain wall and the propagation of these waves in the ferromagnetic domains, micromagnetic simulations are performed. The domain wall is excited by an ac spin-polarized current^26^,^27^, which via the spin-transfer torque effect results in driven domain wall oscillations in a narrow harmonic pinning potential. Regular back-and-forth motion of the magnetic domain wall leads to the emission of spin waves with corresponding frequency. Variation of the driving frequency enables versatile tuning of the spin wave wavelength from the micrometer length scale down to about 100 nm in continuous ferromagnetic layers. Monochromatic excitation of spin waves with even shorter wavelengths is realized by patterning of the ferromagnetic film into magnetic nanowires.

## Results

**Figure 1:**
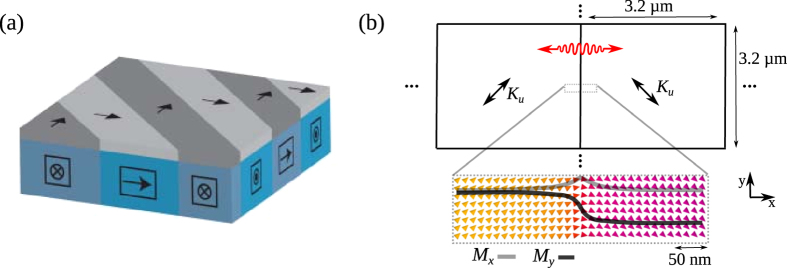
(**a**) Schematic of the ferroelectric-ferromagnetic bilayer structure. The polarization of the ferroelectric *a*_1_ and *a*_2_ domains is aligned along the elongated in-plane axis of the tetragonal BaTiO_3_ lattice and it rotates by 90 degrees at the domain boundaries. Via strain transfer and inverse magnetostriction, uniaxial anisotropy is induced in the ferromagnetic layer. The resulting ferromagnetic domain pattern fully correlates with the ferroelectric domain structure and magnetic domain walls are strongly pinned onto the ferroelectric domain boundaries by abrupt 90 degree rotations of magnetic anisotropy. (**b**) Micromagnetic simulation geometry for an extended film. The computational area is restricted to one period of the stripe domain structure, i.e. a single *a*_1_ and *a*_2_ domain, both having a width of 3.2 *μ*m. Periodic boundary conditions in the *x*- and *y*-direction are used to account for the continuous film geometry. The simulated magnetization pattern near the magnetic domain wall is shown as inset.

Micromagnetic simulations are performed using MuMax3^28^ (see Methods for details). The simulation geometry, which is restricted to a 6.4 × 3.2 *μ*m^2^ region with an *a*_1_ and *a*_2_ domain, is shown in Fig. 1(b). Periodic boundary conditions are applied in both the *x*- and *y*-direction to mimic stripe domains with infinite stripe length (along *y*) and a width of 3.2 *μ*m. Strain coupling to the ferroelectric layer is implemented by a magnetic anisotropy (*K*_*u*_) and abrupt 90 degree rotation of the uniaxial anisotropy axis at the *a*_1_ − *a*_2_ domain boundary. Simulations are performed for zero applied magnetic field after initialization of a head-to-tail magnetization configuration in the ferromagnetic domains (Fig. 1(a)). The width of the magnetic domain walls is of the order 10–100 nm, depending on magnetic anisotropy strength. Abrupt rotation of the magnetic anisotropy boundary ensures strong pinning of perfectly straight magnetic domain walls, in agreement with experimental observations^20^,^21^,^22^,^23^. The domain wall oscillations and spin waves that we consider in this paper only correspond to small perturbations 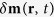 of the equilibrium magnetization state 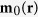. Since the effects of such small 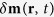 on the polarization state in the ferroelectric layer are negligible, it is possible to simulate the main physical phenomena by considering the ferromagnetic layer only.

### Magnetic domain wall oscillations

**Figure 2:**
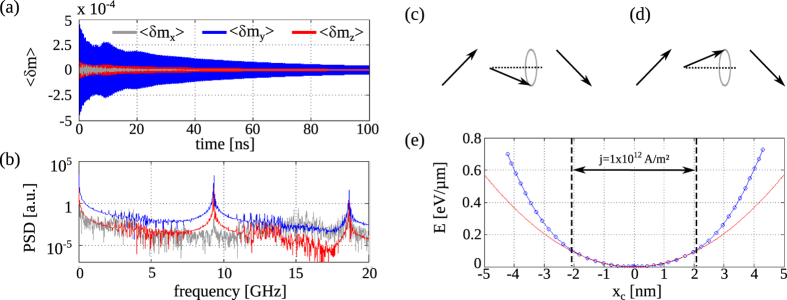
(**a**) Time evolution of the magnetic pertubation 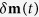 averaged over a 400 nm wide region around the domain wall after the application of a 1 ns current pulse. (**b**) PSDs of the curves presented in (**a**). (**c**,**d**) Sketch of magnetization precession inside the domain wall. At resonance, the magnetic domain wall oscillates back and forth (displacement to the left in (**c**) and to the right in (**d**)). (**e**) Shape of the pinning potential. Symbols are numerical data and the red line is calculated using 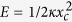, with *κ* and 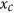 indicating the domain wall stiffness and wall displacement along the *x*-axis. For current densities 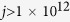 A/m^2^, the pinning potential becomes increasingly anharmonic.

The magnetic domain wall can be described as a quasiparticle that is trapped by a pinning potential. To characterize domain wall pinning under dynamic excitation, we consider a 15 nm thick ferromagnetic layer with saturation magnetization 
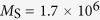
 A/m and exchange constant 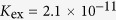 J/m. These values correspond to recent experiments on strain-coupled CoFe films on BaTiO_3_^20^,^21^. The resonant frequency of the pinned domain wall is determined by the application of 1 ns rectangular spin-polarized current pulses with density 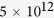 A/m^2^ perpendicular to the domain wall and simulation of the dynamic magnetic response for a subsequent period of 0.1 *μ*s. Via the spin-transfer-torque effect, the current pulses affect the non-uniform magnetization profile of the magnetic domain wall. The time evolution of the perturbations 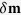 averaged over a 400 nm wide region around the domain wall and the corresponding power spectrum densities (PSDs) are plotted in Fig. 2(a,b). In these simulations, 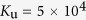 J/m^3^. The PSDs of the averaged magnetization components 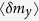 and 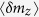 clearly evidence the presence of a resonance at 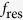
=9.33 GHz. Since the resonance peak is significantly smaller in the PSD of 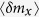, the dynamic mode corresponds to local magnetization precession around the *x*-axis, i.e. perpendicular to the domain wall, as illustrated in Fig. 2(c,d). This rotation of magnetization inside the domain wall corresponds to a damped back-and-forth motion of the wall. Higher harmonics of this oscillation mode are also present, as indicated by the resonance peaks at 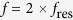 in the PSDs of Fig. 2(b).

To quantify the pinning potential, a microwave spin-polarized current with 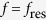 and gradually increasing amplitude is applied perpendicular to the magnetic domain wall. This actuation scheme results in driven domain wall oscillations at the same frequency and increasing oscillation amplitude (see Methods for details). For this dynamic resonance mode, the shape and magnitude of the domain wall pinning potential is determined by the dependence of the system’s potential energy on the position of the wall. A result for 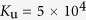 J/m^3^ is shown in Fig. 2(e). Here, the position of the domain wall center *x*_*c*_ (defined as 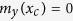) is plotted every time the domain wall reaches the maximum displacement. From the data, a potential stiffness of *κ* = 7.11 × 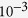 J/m^2^/*μ*m is extracted by fitting a parabolic energy profile 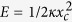 to the simulated curve. Only for small domain wall displacements excited with current densities 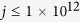 A/m the oscillator is harmonic. A domain wall mass is derived from Mass = 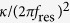. For the data in Fig. 2 this results in a mass of 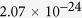 kg per *μ*m length of the magnetic domain wall. This value is in line with domain wall masses of 6.6 × 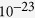 kg, 2.3 × 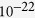 kg, and 1.3 × 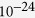 kg, measured on 45 nm × 70 nm, 20 nm × 400 nm, and 20 nm × 2*μ*m wide Permalloy nanowires, respectively^26^,^29^,^30^. Moreover, good fits of the domain wall magnetization profile are obtained using 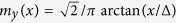 (see Fig. 1). The fitting parameter Δ in this equation is proportional to the domain wall width.

**Figure 3:**
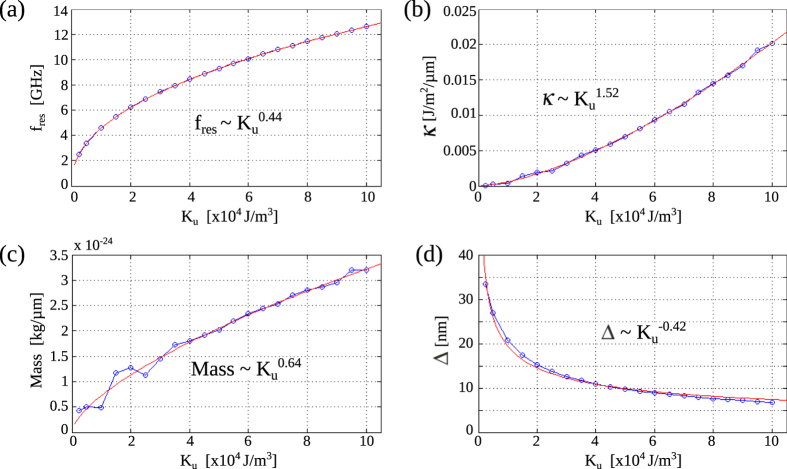
(**a**–**d**) Resonance frequency 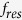, potential stiffness *κ*, magnetic domain wall mass, and domain wall fitting parameter Δ versus magnetic anisotropy strength 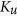. While the data points represent numerical data, the full lines are best fits 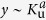.

Using the same methodology, we determined the resonance frequency, potential stiffness, domain wall mass and Δ for different anisotropy strengths 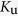. In practice, the latter can be controlled by tuning of the magnetoelastic properties of the ferromagnetic material or variation of the coupling efficiency to the ferroelectric layer. The numerical results, which fully quantify the properties of the magnetic domain wall oscillator, are summarized in Fig. 3. For decreasing 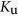, both the domain wall and the pinning potential broaden. Here, 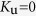 is a singularity as it represents decoupling between the ferromagnetic and ferroelectric layers. In this case, no magnetic domains and domain walls are present and the mass and resonance frequency are zero. The fitted power behaviors agree well with the predictions of a 1-dimensional model that we developed for a pinned 90 degree magnetic domain wall: 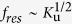, 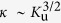, Mass ∼ 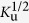, and 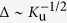 (see Methods).

### Spin wave emission

**Figure 4:**
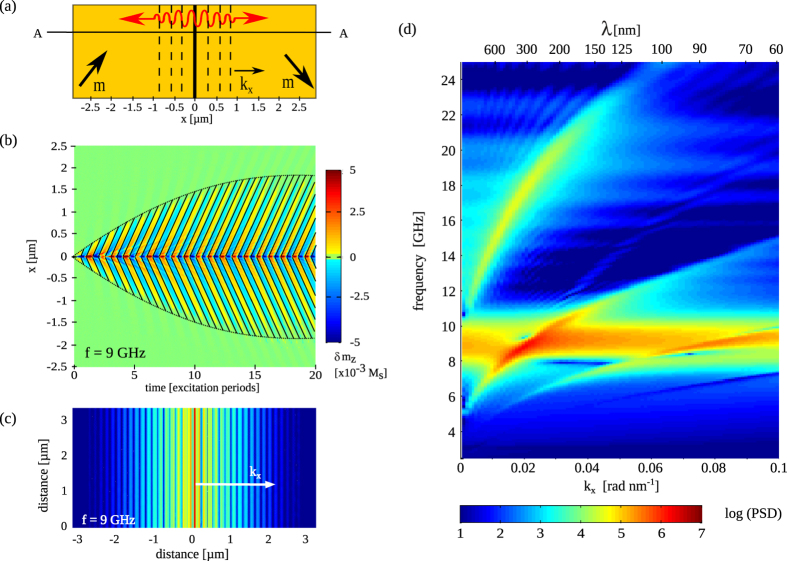
(**a**) Schematic illustration of spin wave emission from an infinitely long oscillating domain wall. The wave fronts of propagating spin waves in the domains are parallel to the domain wall. (**b**) Spin wave profiles along line A in panel (**a**) as a function of excitation time. Solid black lines indicate the location of spin wave nodes. Dotted lines denote the location where the spin wave amplitude drops below 0.15 × 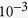 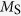. The excitation frequency is 9 GHz. (**c**) Logarithmic plot of the spin wave profile after 20 excitation cycles. (**d**) Dispersion graph of the emitted spin waves after 20 excitation periods. All data were obtained for 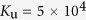 J/m^3^.

When magnetic domain wall oscillations are excited by a microwave spin-polarized current, energy is continuously pumped into the system. During each excitation cycle, the amount of energy furnished to the system is dissipated by the emission of spin waves and internal damping. To investigate the properties of the spin waves, we performed simulations using a sinusoidal spin-polarized current with frequency 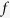 and a density of 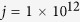 A/m^2^. Because of the infinite length of the magnetic domain wall, the emitted spin waves propagate perpendicular to the domain wall despite the 45 degree angle between the magnetization of the domains and wave vector 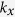 (see next section for more information). In other words, the wave fronts of the spin waves are parallel to the domain wall, as illustrated in Fig. 4(a). Figure 4(b) shows how spin waves with 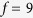 GHz propagate into the ferromagnetic domains. The solid black lines denote the location of spin wave nodes. They illustrate how the two nodes that are generated during each excitation period propagate away from the domain wall. The slope of the lines corresponds to the phase velocity of the spin waves. The dotted lines in Fig. 4(b) indicate the distance from the magnetic domain wall at which the spin wave amplitude drops below 0.15 × 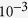 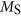. While this envelope is initially determined by the product of phase velocity and time, it converges to a constant value after about 15 excitation periods. Beyond this excitation time, the energy that is continuously pumped into the system by microwave excitation of the oscillating domain wall is balanced by energy dissipation through intrinsic spin wave damping^31^. The out-of-plane magnetization profile after 20 excitation periods is shown in Fig. 4(c). From such a profile, the wave-vector spectrum at excitation frequency 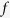 can be extracted by a spatial (1D) Fourier transformation, i.e. 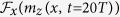. By repeating this simulation approach for different frequencies, a full dispersion diagram (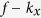) is obtained. A result for 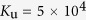 J/m^3^ and frequencies ranging from 2.5 GHz to 25 GHz is shown in Fig. 4(d). Various dispersion bands are observed. The broad horizontal and thus non-dispersive band around 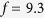 GHz corresponds to the fundamental domain wall oscillation mode. To interpret the other dispersive spin wave bands, the anisotropic nature of spin wave propagation in ferromagnetic domains needs to be taken into account. This is the topic of the next section.

### Characterization of spin wave propagation

**Figure 5:**
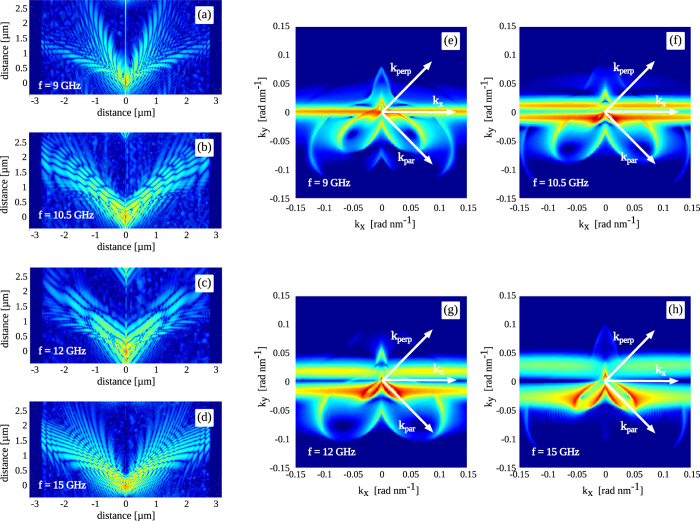
(**a**–**d**) Logarithmic plots of the spin wave profile after local excitation of the domain wall over a length of 50 nm with a sinusoidal spin-polarized current. The plots in (**a**–**d**) are obtained for an excitation frequency of 9 GHz, 10.5 GHz, 12 GHz, and 15 GHz, respectively. (**e**–**h**) Corresponding reciprocal space plots. The horizontal bands represent spin waves that propagate along the domain wall. Other isofrequency contours with wave vectors 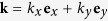 indicate spin waves that propagate into the ferromagnetic domains. In all plots, the color scale ranges over five orders of magnitude.

Spin wave properties depend sensitively on the angle between the wave vector and the direction of magnetization. In most studies, large magnetic bias fields are used to saturate the magnetization throughout the sample. Two main propagation modes are often considered, namely parallel (backward volume spin waves) and perpendicular (surface spin waves) to the magnetization. In the ferromagnetic domains of the hybrid bilayer that we consider, the remanent magnetization direction is set by the uniaxial magnetic anisotropy. The oscillating domain wall emits spin waves at an angle of 45 degrees with respect to this magnetization direction. To obtain further insights in the propagation of spin waves inside the ferromagnetic domains, we interpret the domain wall emitter as a line source that consists of an infinite amount of point sources. Each point along the oscillating domain wall emits spin waves which interfere to an overall spin wave pattern following the Huygens-Fresnel principle. In the following simulations, we analyze the anisotropic propagation properties of spin waves that are emitted from a point-like source by exciting the magnetic domain wall over a length of only 50 nm. Magnetization profiles after 20 excitation periods are shown in Fig. 5 for different frequencies and 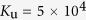 J/m^3^ along with their two-dimensional (2D) wave-vector spectra (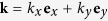), which are obtained by a 2D spatial Fourier transformation of the magnetization profiles, i.e. 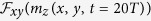.

The simulations reveal that spin waves propagate along the domain wall and into the ferromagnetic domains. At 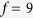 GHz (Fig. 5(a)), the magnetization along the entire domain wall oscillates in phase. The wavelength of the spin waves propagating along the wall is thus infinite and 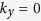. This oscillation mode corresponds to the fundamental resonance of the pinned domain wall. At higher frequencies (Fig. 5(b–d)), nodal lines appear along the domain wall, indicating a decrease of spin wave wavelength and thus an increase of 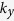. In reciprocal space, spin waves that propagate along the domain wall are reflected by horizontal bands (Fig. 5(e–h)), one at 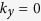 for 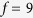 GHz and two symmetric bands at 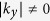 for higher excitation frequencies.

Spin wave propagation into the ferromagnetic domains is highly anisotropic (Fig. 5(a–d)). It is most efficient perpendicular to the local magnetization, i.e. for surface spin waves. In this direction, the wavelength of the spin waves and their decay length are relatively large. At the same frequency, spin waves that propagate parallel to the magnetization (backward volume spin waves) have shorter wavelength and they are attenuated more quickly. With increasing excitation frequency, the wavelengths of both types of spin waves decrease. These observations are also illustrated by the reciprocal space plots of Fig. 5(e–h). The highly anisotropic nature of spin wave propagation inside the ferromagnetic domains is reflected by the congruent shapes of the isofrequency contours. The contours with large wave vector in the third and fourth quadrant correspond to modes with small angles between the wave vector and local magnetization, whereas the first and second quadrant depict modes with large angles. The congruency of the modes and the periodicity in reciprocal space (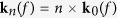 (with *n* an integer)) suggests that modes with 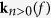 are higher harmonics of the spin wave mode characterized by 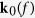.

**Figure 6:**
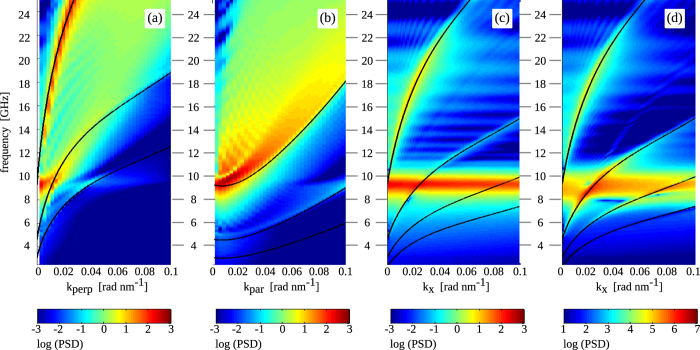
(**a**–**c**) Spin wave dispersion diagrams obtained by exciting the magnetic domain wall over a length of only 50 nm. Spin waves with wave vectors 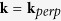 (surface spin waves), 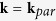 (backward volume spin waves) and 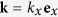 are considered. The black lines in panels (**a**–**c**) are numerically calculated using 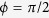, 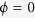, and 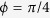, respectively, in equation (1). Harmonics are also included. (d) Dispersion diagram for spin waves that are generated by excitation of the entire magnetic domain wall (the data are identical to those of Fig. 4(d)).

We now consider three specific spin wave propagation directions; parallel and perpendicular to the magnetization inside the domains and perpendicular to the magnetic domain wall, which are indicated by 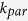, 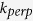 and 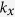 in Fig. 5. A dispersion diagram for each of these spin wave modes is constructed by combining simulation data with different excitation frequency. The results are shown in Fig. 6 (a–c). For further analysis of the spin wave properties, we use the theoretical model of ref. 32 to calculate spin wave dispersion curves for arbitrary angles *ϕ* between the wave vector and local magnetization. In this model, the 0th order approximation for *ω* (=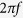) is given by


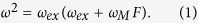


Here


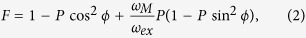



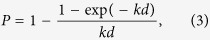


*d* is the sample thickness, 
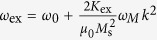
, 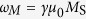, and 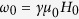. 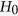 is the total internal field, which corresponds to the anisotropy field in our system. The numerically obtained dispersive branches are indicated by solid lines in Fig. 6. A good match between theory and micromagnetic simulations is obtained for 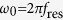, with 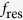 the resonance frequency of the domain wall oscillator. The low-intensity dispersion branches correspond to harmonics 
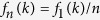
 (with *n* an integer), which originate from the congruent isofrequency contours in reciprocal space. The dispersion diagram for spin wave propagation along the *x*-direction (Fig. 6(c)) contains identical branches as that of Fig. 4(c), which is replotted in Fig. 6(d) for direct comparison. In the latter, the domain wall was excited over its complete length. In the Huygens-Fresnel interpretation, this means that all point sources along the domain wall interfere such that only waves with 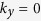 contribute constructively to the resulting spin wave pattern. In addition to these branches, the non-dispersive band centered around the domain wall resonance frequency is also observed.

## Discussion

**Figure 7:**
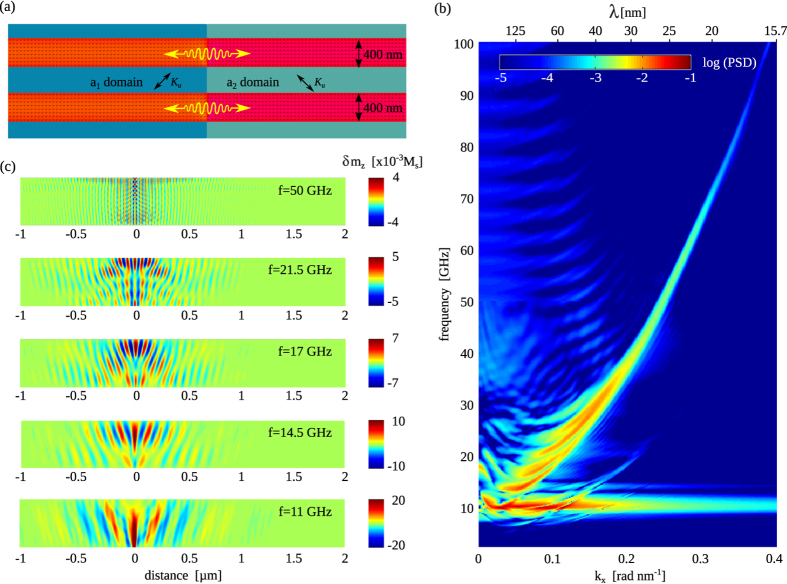
(**a**) Schematic of spin wave excitation and propagation in two parallel magnetic nanowires on top of a ferroelectric layer with an *a*_1_ and *a*_2_ domain. Small arrows indicate the direction of magnetization in zero magnetic field. (**b**) Dispersion graph of propagating spin waves in the nanowires. (**c**) Spin wave profiles for several resonance frequencies of the domain wall oscillator. The profiles are a superposition of spin waves generated by the corresponding *n*th-DE resonance mode at the excitation frequency and some lower order DE modes.

The thin-film domain wall oscillator that we described thus far generates spin waves with wavelengths down to 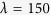 nm and frequencies up to 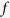 = 24 GHz (see Fig. 4(d)). At higher frequencies, the magnetic domain wall can no longer follow the microwave excitation signal and spin wave emission into the extended film is drastically diminished. Many magnonic devices, however, rely on the ability to excite and guide spin waves through patterned geometries, in particular magnetic nanowires. In most devices, spin waves are generated by microwave antennas and large magnetic bias fields are utilized to saturate the magnetization, guaranteeing uniform propagation along the wire. To investigate how such a nanowire geometry affects spin wave emission from a pinned magnetic domain wall in a ferroelectric-ferromagnetic bilayer, we performed additional micromagnetic simulations. The main findings for two parallel nanowires with a width of 400 nm and a ferromagnetic film thickness of 15 nm are summarized in Fig. 7. The magnetization in the nanowires is fixed by strong coupling to the ferroelectric layer (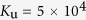 J/m^3^), which makes the application of a bias field superfluous. The dispersion diagram of Fig. 7(b) contains several interesting features. The amplitude of the excitation current is set to 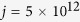 A/m^2^ to increase the spin wave signal. Similar to the extended film geometry, a broad non-dispersive band is obtained around 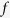 = 11 GHz. As discussed before, this mode stems from resonant back-and-forth motion of the magnetic domain wall. Because of the finite nanowire width, however, additional domain wall resonances appear at higher frequencies. Since the magnetization in the center of the domain wall is oriented perpendicular to the wall, the new modes correspond to Damon-Eshbach-like standing waves (DE modes), i.e. surface spin waves excited in the magnetic domain wall (see Fig. 7(c)). The resonance modes are characterized by the number of nodes along the domain wall at 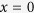. The resonance at 11 GHz can thus be interpreted as the 0th-DE resonance, while higher order resonances at 14.5 GHz, 17 GHz, 21.5 GHz and 25 GHz in the dispersion diagram of Fig. 7(b) can be labeled as 1st-DE, 2nd-DE, 3rd-DE and 4th-DE modes. At resonance, the relation between the standing wave wavelength (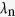) of the *n*th-DE mode and the nanowire width (*w*) is given by 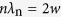. The energy that is efficiently absorbed from the microwave current under these matching conditions is dissipated by the domain wall oscillator via the emission of large-amplitude spin waves into the nanowire. For (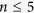), the excitation of spin waves by the different DE modes produces separate bands in the dispersion diagram of Fig. 7(b). Yet, at larger wave vector and frequency, the dispersion of emitted spin waves by higher order DE modes converges onto a single curve. From the slope of this curve, a large spin wave group velocity of 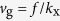 ≈ 2000 m/s is inferred.

Most interestingly, the dispersion diagram evidences that the implementation of a domain wall oscillator in a magnetic nanowire allows for the emission of short-wavelength spin waves at high frequencies. For example, the wave vector of propagating spin waves at an excitation frequency of *f* = 50 GHz in Fig. 7(b) corresponds to *λ* = 25 nm. The reason for these attractive emission properties is twofold: (i) Contrary to the extended film geometry, higher order domain wall resonances exist at high frequency because of spin wave confinement perpendicular to the nanowire axis. As a result, large-amplitude spin waves are emitted into the nanowire over a wide range of excitation frequencies. The spin wave profiles of Fig. 7(c) clearly illustrate this. At *f* = 50 GHz, for instance, the 12th-DE resonance mode generates short-wavelength spin waves that propagate into the nanowire over several micrometers. (ii) The second key feature of the magnetic domain wall oscillator is its straight and narrow excitation area, which is provided by strong pinning onto the few nanometers wide *a*_1_ − *a*_2_ domain boundary in the ferroelectric layer. The uniformity of the pinning potential is thus not limited by the resolution of a lithography process. These important attributes and the absence of long-range magnetic bias fields open up perspectives towards the development of miniaturized magnonic devices. In such sub-micrometer magnonic elements, damping of spin waves is no longer a limiting factor, while large group velocities guarantee fast signal throughput in a broad GHz frequency range.

An important feature of the nanowire oscillator is the monochromatic nature of excited spin waves at high frequency. For instance, the wave vector line width at *f* = 50 GHz is *δk* ≈ 0.01 rad/nm, which corresponds to a wavelength distribution of only *δλ* ≈ 1 nm. Moreover, domain wall oscillators in parallel nanowires that are pinned onto the same ferroelectric domain wall (Fig. 7(a)) emit spin waves with identical dispersion properties. Thus, when the same microwave current is fed into multiple wires, the spin wave sources will emit coherently. If desired, fixed phase relations between propagating spin waves in different nanowires can be attained by tuning the phase of the individual excitation signals. Monochromatic and coherent emission of spin waves from different sources can be used to realize Mach-Zehnder type logic elements and sensors^6^,^33^,^7^. Also, because ferroelectric domains are separated by parallel and perfectly straight domain boundaries it is possible to define identical domain wall oscillators in one-and-the-same magnetic nanowire (contrary to the single oscillator in Fig. 7(a)). Such coherent spin wave sources would allow for the engineering of magnonic band gaps without additional patterning^34^, again alleviating lithographic restrictions.

To summarize, based on micromagnetic simulations, we have proposed a highly tunable source of short-wavelength spin waves. The source consists of a pinned magnetic domain wall that is strain-coupled to a straight and narrow domain boundary in a ferroelectric layer. Oscillations of the magnetic domain wall in the highly localized pinning potential are driven by a microwave spin-polarized current via the spin-transfer torque effect. The energy that is continuously pumped into the domain wall oscillator is dissipated by internal damping and the emission of spin waves at the same frequency as the excitation signal. The properties of the spin wave source depend on the domain wall resonance modes, which can be engineered by patterning of the ferromagnetic layer. In magnetic nanowires, the fundamental back-and-forth oscillation mode is complemented by multiple resonances that are caused by Damon-Eshbach-like standing waves inside the domain wall. As a consequence, pinned domain walls in magnetic nanowires allow for spin wave emission up to about 100 GHz. The wavelength of the excited spin waves at high frequency is uniquely defined (monochromatic emission) and short, typically 10-100 nm, opening new avenues for the realization of nanoscale magnonic devices.

## Methods

### Micromagnetic simulations

The time evolution of the magnetization **m** in the ferromagnetic layer is governed by the Landau-Lifshitz-Gilbert equation^35^ with additional spin-transfer-torque terms to account for the effect of a current density **j**^36^


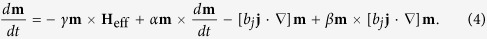


Here, *γ* is the gyromagnetic ratio, *α* is the damping constant and 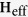 is the effective magnetic field which contains contributions from the exchange, demagnetizing and anisotropy fields. Strain coupling to the ferroelectric layer is taken into account via the magnetic anisotropy field. In our simulations, we vary the strength of the induced anisotropy 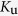 between 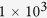 J/m^3^ and 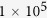 J/m^3^ which, in practice, depends on the magnetoelastic properties of the ferromagnetic layer and the efficiency of strain transfer. Furthermore, in expression (4) 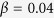 is the degree of non-adiabaticity and 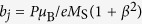 expresses the coupling strength between the applied current and the magnetization (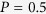 is used as the polarization of the spin-polarized current, *e* is the electron charge and 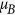 is the Bohr magnetron). Micromagnetic simulations are performed using MuMax3^28^. The ferromagnetic domains are discretized using finite difference cells with sizes 3.125 × 3.125 × 15 nm^3^. While the induced anisotropy strength 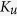 is varied, other material parameters are representative for experiments on CoFe^21^: exchange constant 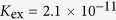 J/m and saturation magnetization 
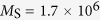
 A/m. In simulations with an extended film geometry, periodic boundary conditions are applied in both the *x*- and *y*-directions. For the magnetic nanowire, this is only done in the *x*-direction. In all simulations, a large damping constant of 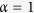 is used in the outer left and right 400 nm wide regions of the simulation area to exclude interference with spin waves that are generated by other domain walls. In the central part of the simulations, *α* is adjusted to give the best possible analysis of different spin wave properties: to determine 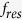 with optimal resolution we used 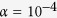, which resulted in large spin wave signals on a 100 ns time scale (see Fig. 2(a)); to ensure that all energy increases in Fig. 2(e) can be attributed to oscillations of the magnetic domain wall, we instantly damped the emitted spin waves using 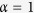; to compute the spin wave dispersions, we used a physically realistic damping parameter of 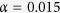.

To calculate the stiffness of the pinning potential, a sinusoidal current density profile with gradually increasing amplitude, 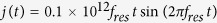, is applied to the domain wall. Here, 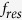 is the resonance frequency of the domain wall oscillator. This actuation scheme results in driven domain wall oscillations with increasing amplitude. The domain wall displacement is collected at every 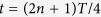, with 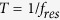 and *n* an integer. This is when the domain wall displacement is maximal and the domain wall velocity is zero. This way, dynamic energy contributions due to domain wall tilting at non-zero domain wall velocities do not affect the data. The obtained energy versus displacement relation (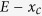) can be fitted by 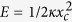, where *κ* is the potential stiffness.

For the simulation of the dispersion graphs of the thin-film geometry, Figs 4(d) and 6(d), a current density 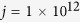 A/m^2^ is imposed to the ferromagnetic layer, which is in the linear region of the oscillator (see Fig. 2(e)). For all other simulations, a higher current density of 5 × 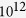 A/m^2^ is imposed. Tests on the thin-film geometry indicate that the use of this higher current density results in an increased power density, but that the anharmonic potential does not excite non-linear spin wave modes. The high current densities are motivated by numerical arguments. The Graphics Processing Units hardware that was used in this work has a 6-digits resolution. As a result, magnetic perturbations of 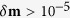 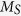 can be resolved. As the emitted spin waves are still linear perturbations, the spin wave intensity scales down linearly with decreasing current density. Less intense spin waves can easily be measured using available experimental techniques^37^,^38^.

### Model of 90 degree magnetic domain wall oscillator

In addition to micromagnetic simulations, we developed a 1-dimensional model to describe the driven oscillations of a 90 degree magnetic domain wall. Again, we consider an anisotropy boundary at 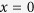. In the left domain (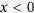) the angle of the anisotropy axis with the *y*-axis is *π*/4, while in the right domain (*x* > 0) it is 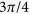 (same as in Fig. 1(b)). We also assume that the magnetic domain wall moves as a rigid structure in the potential well when driven by the spin transfer torque. This assumption allows us to describe the domain wall dynamics using two independent collective variables; the position of domain wall center, 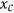, and the out-of-plane domain wall tilt angle, 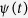.

We express the local magnetization in spherical coordinates, 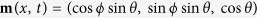. The equilibrium magnetization for each domain can be calculated analytically using the LLG equation. It can be shown that the in-plane domain wall profile is well approximated by a modified Walker ansatz^39^,


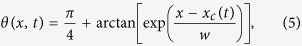


where the domain wall width is given by 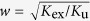. In turn, the out-of-plane domain wall profile is approximated by


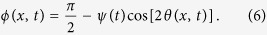


By linearizing the LLG equation, we then obtain linear equations of motion for the collective coordinates 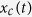 and 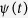 of the form


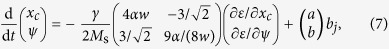


where *ε* is the areal energy density 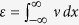, with *ν* being the volume energy density. Moreover, 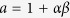, and 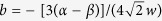. Solving Eq. 7 in the linear response approximation one obtains the resonance frequency of the domain wall


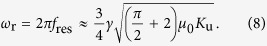


From the definition of potential stiffness one obtains 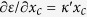, where 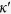 is the potential stiffness per unit area. Using the linearized model this gives 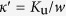, which indicates that 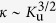. Finally, taking into account the results for potential stiffness and resonance frequency one finds that the domain wall mass changes as Mass ∼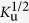. The predictions of the linearized 1-dimensional model are in good agreement with the micromagnetic simulations (Fig. 3).

## Additional Information

**How to cite this article**: Wiele, B. V. *et al.* Tunable short-wavelength spin wave excitation from pinned magnetic domain walls. *Sci. Rep.* **6**, 21330; doi: 10.1038/srep21330 (2016).
